# The international legal framework for transporting infectious substances

**DOI:** 10.2471/BLT.25.295244

**Published:** 2026-09-09

**Authors:** Markus Huber, Ekin Sahin, Lance D Presser, Kazunobu Kojima, Joanna Salvi Le Garrec

**Affiliations:** a Emerging Threat Diagnostics and Laboratory Biosafety, Epidemic and Pandemic Management, WHO Health Emergencies Programme, World Health Organization, Avenue Appia 20, 1211 Geneva 27, Switzerland.; b Passenger and Cargo Control Programme, United Nations Office on Drugs and Crime, Vienna, Austria.; c Centre for Infectious Diseases Research, Diagnostics and Laboratory Surveillance, National Institute for Public Health and Environment, Bilthoven, Kingdom of the Netherlands.; d Health Security Division, WHO Regional Office for Europe, Copenhagen, Denmark.

## Abstract

**Objective:**

To examine international agreements, standards, policy documents and regulations governing the cross-border transportation of infectious substances and to assess the extent to which fragmented regulatory frameworks affect timely and safe shipment.

**Method:**

A qualitative document analysis of 30 binding and non-binding legal and policy instruments was conducted and details were entered into a common extraction grid. In addition, the published reports and 64 international participants of World Health Organization (WHO) subregional workshops on the transport of infectious substances legislation, in Istanbul, Türkiye (2022) and Almaty, Kazakhstan (2023), provided an operational perspective. No other interviews or consultations were undertaken.

**Findings:**

The analysis revealed substantial fragmentation and inconsistency in regulatory frameworks governing the cross-border transportation of infectious substances. Although many countries had ratified or referenced international agreements in national legislation, harmonized and cross-departmental implementation of legislation was lacking. Limitations included: (i) the incomplete adoption of international shipping standards; (ii) bureaucratic delays in approval and licensing processes; (iii) limited technical capacity; and (iv) a lack of standardized procedures and enforcement mechanisms.

**Conclusion:**

The international regulatory environment for infectious substance transport is complex and targeted interventions are needed, such as harmonizing regulatory frameworks, improving interagency coordination, investing in capacity-building, developing standard operating procedures and aligning policy documents with international best practice. These interventions will help countries meet their obligations under the International Health Regulations (2005), enhance the safe and secure transport of infectious substances and improve global public health preparedness, as required by the recent WHO Pandemic Agreement.

## Résumé

**Objectif:**

Examiner les accords internationaux, les normes, les documents stratégiques et les réglementations régissant le transport transfrontalier de substances infectieuses et évaluer dans quelle mesure la fragmentation des cadres réglementaires affecte la rapidité et la sécurité des expéditions.

**Méthodes:**

Une analyse documentaire qualitative a été menée sur 30 instruments juridiques et politiques, contraignants ou non, et des informations détaillées ont été saisies dans une grille d’extraction commune. En outre, les rapports publiés et les 64 participants internationaux aux ateliers sous-régionaux de l’Organisation mondiale de la Santé (OMS) sur la législation relative au transport des substances infectieuses, organisés à Istanbul (Turquie) en 2022 et à Almaty (Kazakhstan) en 2023, ont apporté un éclairage opérationnel. Aucun autre entretien ni aucune autre consultation n’ont eu lieu.

**Résultats:**

L’analyse a révélé une fragmentation et un manque de cohérence importants dans les cadres réglementaires régissant le transport transfrontalier de substances infectieuses. Bien que de nombreux pays aient ratifié ou fait référence à des accords internationaux dans leur législation nationale, une mise en œuvre harmonisée et transversale de la législation faisait défaut. Parmi les limitations figuraient: (i) l’adoption incomplète des normes internationales de transport; (ii) des retards administratifs dans les procédures d’autorisation et d’octroi de licences; (iii) des capacités techniques limitées; et (iv) le manque de procédures normalisées et de mécanismes de mise en œuvre.

**Conclusion:**

L’environnement réglementaire international régissant le transport des substances infectieuses est complexe et nécessite des interventions ciblées, telles que l’harmonisation des cadres réglementaires, l’amélioration de la coordination interinstitutionnelle, l’investissement dans le renforcement des capacités, l’élaboration de procédures opérationnelles standardisées et l’harmonisation des documents stratégiques selon les bonnes pratiques internationales. Ces interventions aideront les pays à respecter leurs obligations au titre du Règlement sanitaire international (2005), à renforcer la sécurité et la sûreté du transport de substances infectieuses et à améliorer la préparation en matière de santé publique à l’échelle mondiale, comme l’exige le récent Accord de l’OMS sur les pandémies.

## Resumen

**Objetivo:**

Examinar los acuerdos internacionales, las normas, los documentos de política y los reglamentos que rigen el transporte transfronterizo de sustancias infecciosas y evaluar en qué medida la fragmentación de los marcos regulatorios afecta a su envío oportuno y seguro.

**Métodos:**

Se realizó un análisis documental cualitativo de 30 instrumentos jurídicos y de política, vinculantes y no vinculantes, y se registraron los datos en una matriz común de extracción. Además, los informes publicados y las aportaciones de 64 participantes internacionales en los talleres subregionales de la Organización Mundial de la Salud (OMS) sobre la legislación relativa al transporte de sustancias infecciosas, celebrados en Estambul (Türkiye) en 2022 y Almaty (Kazajstán) en 2023, proporcionaron una perspectiva operativa. No se realizaron otras entrevistas ni consultas.

**Resultados:**

El análisis reveló una fragmentación y una falta de coherencia considerables en los marcos regulatorios que rigen el transporte transfronterizo de sustancias infecciosas. Aunque muchos países habían ratificado acuerdos internacionales o habían hecho referencia a ellos en su legislación nacional, faltaba una aplicación armonizada de la legislación entre los distintos departamentos. Entre las limitaciones se encontraban: (i) la adopción incompleta de las normas internacionales de transporte; (ii) las demoras burocráticas en los procesos de autorización y concesión de licencias; (iii) una capacidad técnica limitada; y (iv) la falta de procedimientos normalizados y mecanismos de cumplimiento.

**Conclusión:**

El entorno regulatorio internacional para el transporte de sustancias infecciosas es complejo y se necesitan intervenciones específicas, como armonizar los marcos regulatorios, mejorar la coordinación interinstitucional, invertir en el fortalecimiento de capacidades, elaborar procedimientos operativos normalizados y armonizar los documentos de política con las mejores prácticas internacionales. Estas intervenciones ayudarán a los países a cumplir sus obligaciones en virtud del Reglamento Sanitario Internacional (2005), reforzar la seguridad y la protección en el transporte de sustancias infecciosas y mejorar la preparación mundial en materia de salud pública, tal como exige el reciente Acuerdo de la OMS sobre Pandemias.

## ملخص

**الغرض:**

فحص الاتفاقيات، والمعايير، ومستندات السياسات، واللوائح الدولية التي تتحكم في النقل العابر للحدود للمواد المعدية، وتقييم مدى تأثير أطر العمل التنظيمية المجزأة على عمليات الشحن الآمنة ذات التوقيت المناسب.

**الطريقة:**

تم إجراء تحليل نوعي للمستندات شمل 30 أداة قانونية وسياساتية ملزمة وغير ملزمة، وتم تدوين التفاصيل في نموذج موحد لاستخلاص البيانات. وبالإضافة إلى ذلك، فإن كل من التقارير المنشورة، و64 مشاركاً دولياً في ورش العمل دون الإقليمية التي نظمتها منظمة الصحة العالمية بشأن تشريعات نقل المواد المعدية، والتي عُقدت في إسطنبول بتركيا (2022) وألماتي بكازاخستان (2023)، قد قدمت منظوراً عملياً. ولم يتم إجراء أية مقابلات شخصية أو استشارات أخرى.

**النتائج:**

كشف التحليل عن تجزئة وعدم اتساق كبيرين في أطر العمل التنظيمية التي تتحكم في النقل العابر للحدود للمواد المعدية. ورغم أن العديد من الدول قد قامت بالتصديق على الاتفاقيات الدولية أو أشارت إليها في تشريعاتها الوطنية، إلا أن التنفيذ المنسق والمشترك بين الإدارات لتلك التشريعات كان مفقوداً. وشملت أوجه القصور ما يلي: (1) عدم الاعتماد الكامل لمعايير الشحن الدولية؛ و(2) التأخيرات البيروقراطية في عمليات الموافقة والترخيص؛ و(3) محدودية القدرات التقنية؛ و(4) غياب الإجراءات الموحدة وآليات الإنفاذ.

**الاستنتاج:**

تتسم البيئة التنظيمية الدولية لنقل المواد المعدية بالتعقيد، وتستدعي الحاجة إلى تدخلات موجهة، مثل توحيد أطر العمل التنظيمية، وتحسين التنسيق بين الهيئات، والاستثمار في بناء القدرات، ووضع إجراءات تشغيلية قياسية، ومواءمة وثائق السياسات مع أفضل الممارسات الدولية. وستساعد هذه التدخلات الدول على الوفاء بالتزاماتها بموجب اللوائح الصحية الدولية (2005)، وتعزيز النقل الآمن والمأمون للمواد المعدية، وتحسين استعداد الصحة العامة العالمية، بما يتماشى مع متطلبات اتفاقية الجوائح الأخيرة التابعة لمنظمة الصحة العالمية.

## 摘要

**目的:**

剖析管控跨境运输传染性物质的国际协议、标准、政策文件和法规，评估碎片化管控框架对及时且安全地进行运输的影响程度。

**方法:**

我们针对 30 项具有约束力及无约束力的法律和政策文书开展了一项定性文献分析，相关详细信息已输入统一提取表格。此外，世卫组织 (WHO) 在土耳其伊斯坦布尔（2022 年）、哈萨克斯坦阿拉木图（2023 年）就传染性物质运输立法举办了次区域研讨会，该研讨会发布的报告和 64 名国际参与者提供了一个操作层面的视角。未开展其他访谈或咨询工作。

**结果:**

这项分析揭示了管控跨境运输传染性物质的法规框架中存在非常明显的碎片化与不一致现象。虽然许多国家在本国立法中批准过或引用过相关国际协定，但仍缺少统一协调的跨部门实施。局限性包括：(i) 国际运输标准未被完整采纳；(ii) 审批和许可流程存在行政性延误；(iii) 技术能力有限；且 (iv) 缺少标准化流程和强制实施机制。

**结论:**

传染性物质运输的国际监管环境非常复杂，需要采取针对性的干预措施，例如统一监管框架、提升机构间的协作、对能力建设进行投资、制定标准的操作流程，并使政策文件与国际最佳实践保持一致。这些干预措施将帮助各国根据《国际卫生条例（2005 年）》履行其义务，提升传染性物质的安全与可靠运输，按最近的《世卫组织大流行协定》的要求提高全球公共卫生的应对准备能力。

## Резюме

**Цель:**

Изучить международные соглашения, стандарты, программные документы и нормативные акты, регулирующие трансграничную перевозку инфекционных веществ, и оценить, в какой степени фрагментарность нормативно-правовой базы влияет на своевременность и безопасность доставки.

**Методы:**

Проведен качественный анализ 30 обязательных и необязательных правовых и программных документов, сведения из которых были внесены в единую таблицу для сбора данных. Кроме того, опубликованные отчеты и материалы, полученные от 64 международных участников субрегиональных семинаров Всемирной организации здравоохранения (ВОЗ) по вопросам законодательства в сфере перевозки инфекционных веществ, проведенных в Стамбуле, Турция (2022 год), и Алматы, Казахстан (2023 год), позволили учесть практический аспект. Других интервью или консультаций не проводилось.

**Результаты:**

Анализ выявил значительную фрагментарность и несогласованность нормативно-правовой базы, регулирующей трансграничную перевозку инфекционных веществ. Хотя многие страны ратифицировали международные соглашения или ссылались на них в национальном законодательстве, отсутствовало согласованное межведомственное внедрение законодательства. Ограничения включали: (i) неполное принятие международных стандартов перевозки; (ii) бюрократические задержки в процессах утверждения и лицензирования; (iii) ограниченные технические возможности; (iv) отсутствие стандартизированных процедур и механизмов обеспечения соблюдения требований.

**Вывод:**

Международная нормативно-правовая среда в сфере перевозки инфекционных веществ является сложной и требует целенаправленных мер, таких как гармонизация нормативно-правовой базы, улучшение межведомственной координации, инвестирование в наращивание потенциала, разработка стандартных операционных процедур и согласование программных документов с передовой международной практикой. Эти меры помогут странам выполнять свои обязательства по Международным медико-санитарным правилам (2005 года), повысить безопасность и защиту при перевозке инфекционных веществ, а также улучшить глобальную готовность систем общественного здравоохранения, как того требует недавно принятое Соглашение ВОЗ по борьбе с пандемиями.

## Introduction

The transportation of infectious substances carries inherent health risks to people, animals and the environment, which means that safe and secure handling is essential for protecting public health. The International Health Regulations (IHR, 2005) obligate Member States of the World Health Organization (WHO) to ensure the safe management and transport of biological substances, diagnostic specimens and materials required for public health, including their entry and exit from territories, their processing and their disposal.^1^

The WHO Pandemic Agreement adopted by the World Health Assembly in 2025 reinforces this obligation and emphasizes strengthened supply chains, well-coordinated logistics and safe, timely transport as core elements of pandemic preparedness (Articles 6, 7, 8 and 13 of the Agreement).^2^ In addition, the Agreement calls for regulatory harmonization, capacity-building and the protection of transport and logistics workers.

To support Member States in their efforts to meet their obligations under IHR, we aimed to identify systemic gaps in legislation and, subsequently, to propose actions for improving regulatory coherence, technical capacity and coordination between national and international agencies. We review the international agreements and standards governing the cross-border transport of infectious substances and clarify key considerations in their dispatch and receipt. Although many states have incorporated international provisions into national law, gaps remain in the implementation of these laws due to bureaucratic delays and insufficient technical capacity.^3^^,^^4^

## Methods

We employed a qualitative research method grounded in the READ (i.e. read, extract, analyse and distil) approach.^5^ To ensure transparency and rigour, the research process was guided by the COREQ (consolidated criteria for reporting qualitative research) framework.^6^

To explore the regulatory landscape governing the cross-border transportation of infectious substances, we conducted a systematic review of legal instruments, international agreements, scholarly literature and official guidelines. The review was informed by challenges and proposed solutions identified during WHO subregional workshops held in Istanbul, Türkiye in November 2022 and Almaty, Kazakhstan in March 2023.^3^^,^^4^ These workshops, which involved 64 representatives of various national authorities, provided insights into real-world gaps in regulation, in coordination between national and international authorities, and in technical capacity. The discussions served as a foundation for framing our review and as a reference point for interpreting the international policy environment.

No interviews, focus groups or consultations were conducted beyond these two workshops. Consequently, the qualitative material we analysed came from only two sources: (i) published reports of the Istanbul and Almaty workshops, which recorded the plenary discussions, country presentations and structured group work of the 64 participating officials;^3^^,^^4^ and (ii) a corpus of legal and policy documents. The COREQ framework was applied to the reporting of the workshop-derived material rather than to primary interview data.

The document corpus was assembled in three steps. First, the binding and non-binding legal and policy instruments named by participants during the two workshops were listed. Second, that list of instruments was extended through searches of the treaty and standards repositories of the United Nations (UN), WHO, the International Civil Aviation Organization, the International Maritime Organization, the Intergovernmental Organisation for International Carriage by Rail, the Universal Postal Union, the World Customs Organization, the World Trade Organization, the World Organisation for Animal Health (WOAH), the Food and Agriculture Organization of the United Nations, and the secretariats of the Convention on Biological Diversity and the Convention on International Trade in Endangered Species of Wild Fauna and Flora, complemented by searches of the PubMed®, Scopus and Google Scholar databases and of national legal gazettes and government portals. Third, national legal and policy instruments were sampled purposively to illustrate the range of regulatory approaches adopted rather than to achieve exhaustive coverage. Documents were included if they were in force at the time of the review, publicly available in English, French, German or Russian, and contained provisions applicable to the classification, authorization, packaging, movement or import and export of infectious substances. Thirty documents met these criteria and their details were entered into a common extraction grid that covered the scope of the instruments, definitions, the competent authority, substantive obligations, and interactions with other legal regimes. 

We identified policy priorities in the distil step of the READ approach. We mapped themes that recurred in the two subregional workshop reports against gaps in the document analysis, and we retained only those themes that were raised independently in the two workshops and that corresponded to identifiable gaps in the documentary record. All coauthors reviewed and agreed on the resulting priorities, which were also checked for consistency with obligations arising under the IHR (2005) and the WHO Pandemic Agreement.

One author conducted the literature and document review, which focused on international frameworks relevant to infectious substance transportation. Supplementary information was gathered from academic databases, government websites and the official publications of organizations such as WHO, the International Civil Aviation Organization and other UN bodies.

A second author synthesized the data set, which included international agreements, policy documents and workshop insights, using thematic analysis aligned with the READ method. Key patterns, regulatory challenges and best practices were identified, from which a structured and comparative understanding of the international legal and operational landscape was derived.

Although the study aimed to be broadly representative, there was a potential source of bias: the research team’s involvement with WHO-led workshops may have influenced the selection of, and the emphasis on, certain agreements. This familiarity, which provided contextual depth, may also have shaped the scope of the analysis.

## Results

The international transport of infectious substances is regulated through a mosaic of overlapping legal frameworks that address, for example, biosafety, health security, trade, biodiversity and environmental protection (Fig. 1 and Table 1). While these regulatory systems involve different definitions and obligations, their overlapping requirements often create complex and fragmented regulatory environments that complicate national implementation of regulations and cross-border compliance and shipment.

**Fig. 1:**
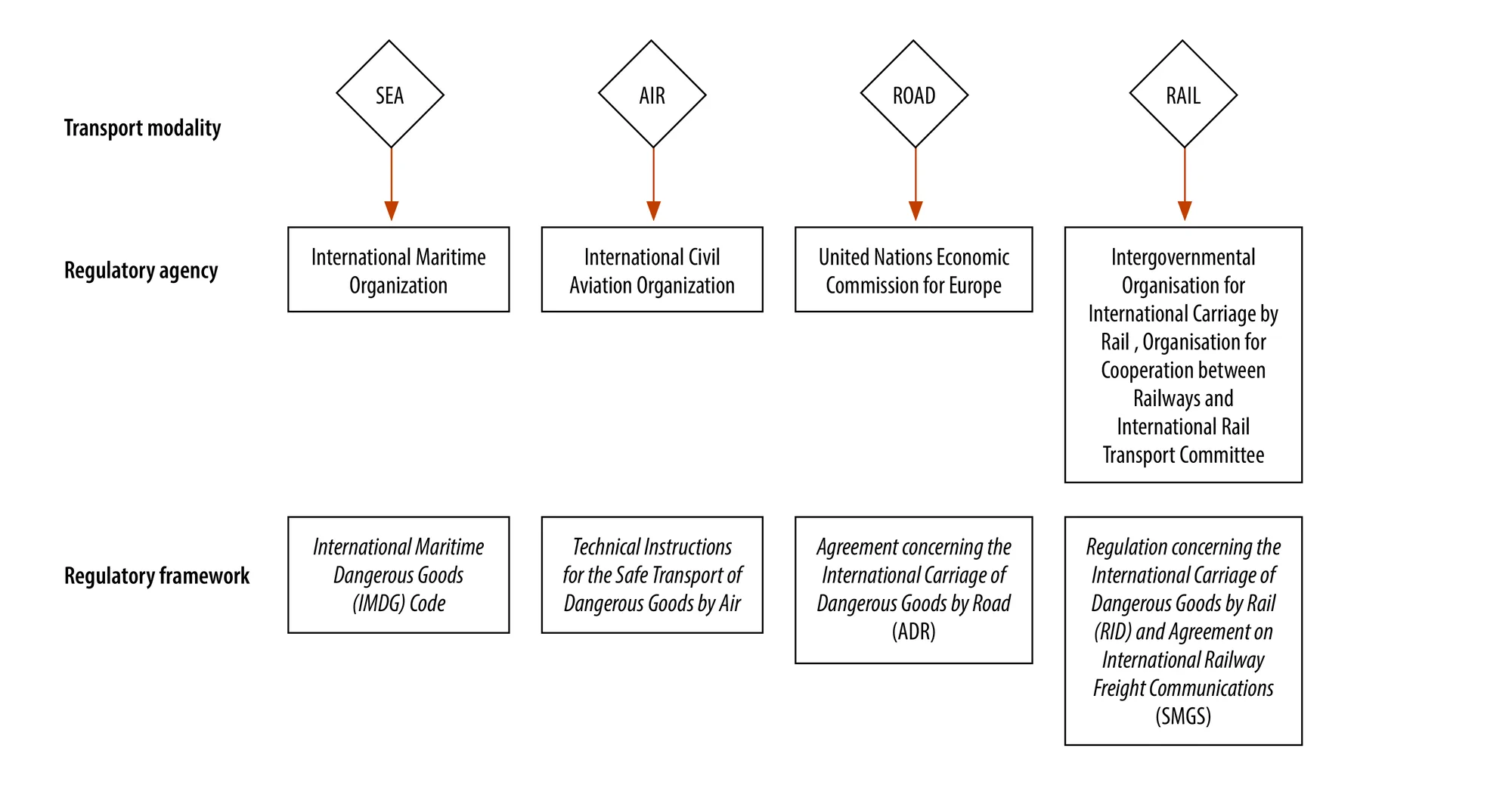
International regulations on transporting infectious substances, by transport modality, 2026

Notes: all transport regulations are underpinned by the United Nations *Model regulations on the transport of dangerous goods*, 24th edition.^7^ The postal system is regulated by the Universal Postal Union through its Convention manual.

**Table 1:** International legal frameworks for transporting infectious substances

Frameworks by category	Relevance to infectious substance transport
**Public health and biosafety**	
IHR (2005);^1^ WHO Pandemic Agreement;^2^ *Guidance on regulations for the transport of infectious substances 2025–2026*^8^	Establish obligations for preparedness, surveillance and safe handling of biological materials, including pathogen sharing, transport procedures, packaging, documentation and personnel training
**Transport safety**	
*Model regulations on the transport of dangerous goods Rev. 24*;^7^ *Technical instructions for the safe transport of dangerous goods by air*;^9^ *Regulation concerning the international carriage of dangerous goods by rail (RID)*;^10^ *International maritime dangerous goods (IMDG) code*;^11^ *ADR Agreement concerning the international carriage of dangerous goods by road*;^12^ *Convention manual* from Universal Postal Union^13^	Regulate infectious substances as dangerous goods and provide requirements for classification, packaging, labelling, documentation and transport by air, sea, rail, road and postal services
**Environmental and biodiversity**	
*Basel Convention on the control of transboundary movements of hazardous wastes and their disposal*;^14^ *Cartagena protocol on biosafety*;^15^ *Nagoya Protocol on access to genetic resources and the fair and equitable sharing of benefits arising from their utilization*;^16^ *Convention on international trade in endangered species of wild fauna and flora*^17^	Regulate the transboundary movement, use and disposal of biological, genetically modified and protected materials, including access, benefit-sharing and biodiversity conservation considerations
**Biosecurity and disarmament**	
*Convention on the prohibition of the development, production and stockpiling of bacteriological (biological) and toxin weapons and on their destruction*;^18^ United Nations Security Council Resolution 1540;^19^ Australia Group Common Control Lists;^20^^–^^22^ Wassenaar Arrangement Control Lists^23^	Prevent the misuse of pathogens and toxins, establish national biosecurity controls and regulate dual-use biological materials that may require export permits
**Animal health and trade**	
*Terrestrial animal health code*;^24^ *FAO biosecurity toolkit*;^25^ Taking a multisectoral one health approach: a tripartite guide to addressing zoonotic diseases in countries;^26^ Regulation (EC) No 1069/2009;^27^ Commission Regulation (EU) No 142/2011^28^	Govern the movement of animal pathogens, animal by-products and zoonotic materials to prevent the spread of animal diseases across borders
**Customs and trade**	
World Customs Organization harmonized commodity description and coding system;^29^ *Agreement on trade facilitation*^30^	Support customs classification, documentation and border clearance procedures and promote coordination among customs authorities to facilitate the timely movement of shipments

FAO: Food and Agriculture Organization of the United Nations; IHR: International Health Regulations; WHO: World Health Organization.

National regulatory frameworks frequently introduce stricter or supplementary measures beyond those required by international standards; for example, mandatory compliance with laws on the national transport of dangerous goods that extend beyond the UN model regulations.^3^^,^^4^

Some countries, such as China,^31^ Kazakhstan^32^ and the Russian Federation,^33^ have developed detailed domestic laws on biosafety and pathogen transport. These laws are aligned with international norms but country-specific requirements for authorization and control have been added.

Countries can apply enhanced classification, packaging or permit requirements, particularly for high-risk pathogens, and can operate import–export licensing systems or notification procedures for transfers of infectious materials. For instance, India regulates the transport of infectious waste and diagnostic material under its biomedical waste management rules,^34^ whereas South Africa enforces additional controls through its Hazardous Substances Act.^35^ Similar authorization frameworks apply in Japan and the United States of America,^36^^,^^37^ where high-consequence agents and toxins are subject to facility registration and shipment notification systems.

In addition, several jurisdictions mandate the registration of facilities authorized to handle infectious substances and the certification of personnel handling them to ensure compliance with biosafety and biosecurity obligations. Examples include Canada’s dangerous goods classification requirements.^38^

Although these measures strengthen national oversight and build domestic capacity, they can complicate cross-border shipments and international cooperation because administrative processes, documentation formats, and licensing and permit processes may differ between competent authorities.

### Overlapping categories and definitions

A single biological material can be governed simultaneously by multiple intersecting legal regimes, each with its own definitions, classification systems and compliance obligations. This regulatory complexity reflects the overlapping goals of transport safety, biosafety, biosecurity, public health protection, environmental safety, biodiversity, trade regulation and national governance.

#### Transport safety

The UN model regulations define infectious substances as materials “known or reasonably expected to contain pathogens” capable of causing disease in humans or animals.^7^ They distinguish infectious substances classified as Category A, which includes high-consequence pathogens such as the Ebola virus, from those classified as Category B, which includes routine diagnostic and clinical specimens, and define exempted materials, thereby guiding packaging, labelling and documentation requirements across all transport modes.

#### Environmental and waste control

The Basel Convention on the Control of Transboundary Movements of Hazardous Wastes extends the definition of hazardous wastes to include infectious wastes from medical, veterinary and research activities. Consequently, prior informed consent is required for their disposal and disposal must be done in an environmentally sound manner.^14^

#### Genetic resources and benefit-sharing

The Nagoya Protocol on access and benefit-sharing treats biological materials containing genetic resources as subject to sovereign rights; hence transfers for research, diagnostics or vaccine development may require prior informed consent by, and mutually agreed terms with, the providing country.^16^

#### Export control, biosecurity and health security

Infectious substances may qualify as dual-use goods if the same material could serve legitimate scientific and public health purposes and could be used for the development of biological weapons: the Australia Group and Wassenaar Arrangement control lists include high-risk pathogens and related technologies that require export authorization.^20^^,^^23^

#### Animal health

Materials containing, or derived from, animal pathogens, tissues or by-products fall additionally under WOAH standards and regional rules,^24^ for example European Union (EU) Regulation (EC) No 1069/2009 and Commission Regulation (EU) No 142/2011,^27^^,^^28^ which set biosafety, traceability and disposal requirements.

#### Customs classification and trade facilitation

Infectious substances fall under customs regulations because of their dual relevance to public health and to security. The World Customs Organization’s Harmonized System classification enables customs officers to identify infectious or diagnostic materials and verify compliance with permit, packaging and documentation requirements under the European *Agreement concerning the international carriage of dangerous goods by road* and the International Civil Aviation Organization’s *Technical instructions for the safe transport of dangerous goods by air*.^9^^,^^12^^,^^29^

#### National import registration and legislation on benefit-sharing and access

Many countries require import permits, facility registration or prior notification for the transfer of infectious or genetically modified materials. National access and benefit-sharing laws that implement the Nagoya Protocol may additionally regulate pathogen-sharing for research or public health responses,^16^ thereby sometimes complicating the rapid investigation of disease outbreaks.

As a result, the same pathogen sample, diagnostic specimen or genetically modified organism may trigger parallel requirements under different international and national regulatory frameworks. For instance, a diagnostic veterinary swab containing *Bacillus anthracis* could concurrently fall under transport safety, biosafety, biodiversity and genetic resource, transboundary waste, animal health, and export control regimes.

### Insights from WHO workshops

At the WHO subregional workshops in Istanbul and Almaty, common regulatory barriers to the safe and timely transport of infectious substances were identified.^3^^,^^4^ Key issues included: (i) the unclear division of responsibilities among competent authorities; (ii) the absence of standardized national procedures; (iii) no harmonized implementation of international transport rules; and (iv) lengthy licensing and customs processes. In several countries, obtaining permits for Category A substances^7^ took two weeks to two months and occasionally led to the cancellation or destruction of shipments.^3^^,^^4^ In addition, reports of frequent misclassifications, inconsistent customs practices and delays highlighted the need for standardized coding, better interagency coordination and biosafety training for customs officials to ensure the efficient and safe cross-border transport of infectious materials.^3^^,^^4^

Additional challenges included limited interdepartmental coordination, a shortage of personnel who were trained and certified in biosafety, biosecurity and customs operations, and regulatory agencies with insufficient technical capacity to assess shipment materials without external experts.^3^^,^^4^ Workshop participants reported widespread shortages of certified couriers, dry ice and UN-certified packaging, and limited access to legal and guidance documents in the local language, particularly Russian.^3^^,^^4^ In addition, financial constraints hindered implementation, with several WHO Member States relying on donor or partner laboratory support for shipment costs.^3^^,^^4^

At both workshops, the veterinary sector was reported to experience the most severe bottlenecks: access to certified couriers was restricted, import permits were inconsistent and packaging supplies were limited.^3^^,^^4^

Other reported barriers included the absence of national classification systems for dangerous goods, incomplete or out-of-date dual-use control lists, and weak biosafety and biosecurity legislation. Moreover, a lack of coordination among ministries and border agencies created duplicative or inconsistent procedures, and non-laboratory health staff often had little awareness of, or no protocols for, safe transport practices. Overall, both workshops revealed the existence of systemic weaknesses, fragmented legislation, procedural ambiguity, insufficient training and infrastructure, limited financing, and weak intersectoral collaboration, all of which resulted in delays, non-compliance with regulations and barriers to the safe and efficient transport of infectious substances.^3^^,^^4^

## Discussion

### Operational and regulatory barriers

To ensure an international shipment assembled from a small number of scarce components is compliant with regulations, the person responsible for the shipment must: (i) classify the material as Category A (i.e. UN 2814 or UN 2900) or Category B (i.e. UN 3373) according to the UN model regulations;^7^ (ii) obtain UN-certified triple packaging that meets packing instruction P620 or P650;^7^ (iii) hold current training and certification under the International Civil Aviation Organization’s technical instructions or the European *Agreement concerning the International Carriage of Dangerous Goods by Road*;^9^^,^^12^ (iv) secure export and import permits and, where applicable, dual-use or access and benefit-sharing authorization;^20^^,^^23^ (v) procure dry ice or a dry shipper with the associated Class 9 documentation, according to UN model regulations;^7^ and (vi) book a carrier and courier willing to accept Class 6.2 cargo.^7^ A single missing element voids the consignment.

The supplier base is narrow: certified packaging is produced by a small number of manufacturers and was rarely stocked domestically in the countries represented at the two WHO subregional workshops; dry ice production is concentrated in capitals; and the number of couriers and airlines accepting Category A cargo is limited to a single operator in several countries.

Where WHO or a partner organization can facilitate transport, this bottleneck is bypassed rather than resolved: for example, the Global Influenza Surveillance and Response System shipping fund project,^39^ the WHO Ebola Shipment Funds Project and the coronavirus disease-2019 (COVID-19) shipment mechanism each contract designated couriers centrally and meet the freight costs, precisely because many national laboratories could not otherwise ship at all.^40^^,^^41^

Participants at the workshops reported similar use cases driving cross-border movement of infectious substances: (i) confirmatory and reference testing of outbreak specimens; (ii) referral of specimens for genomic sequencing, external quality assessment and proficiency testing panels; (iii) supply of reference materials and reagents; and (iv) provision of specimens for collaborative research and product development. Tolerance of delay differs sharply; confirmatory testing during a disease outbreak is of little value if the specimen arrives weeks late, whereas proficiency panel testing can accept a slower schedule – yet the same permit pathway may apply to both uses. In addition, workshop participants reported it could take two weeks to two months to authorize a Category A shipment, with the occasional cancellation or destruction of the consignment in the interim. Costs were reported to fall unevenly: as few health ministries had a budget for shipment and there was a reliance on donor projects or partner laboratories, the ability to send a specimen often depended on whether an externally funded mechanism happened to be active.^3^^,^^4^

To improve the operational side of the international transport of infectious substances, therefore, there is a need for: (i) predictable, time-bound permitting with an emergency permit pathway distinct from the routine pathway; (ii) reliable domestic supply routes for certified packaging and dry ice; (iii) a standing roster of trained and certified shippers, so that capability does not rest in a single individual; and (iv) a national budget for transport, so that outbreak specimens can be moved irrespective of donor cycles.

At both workshops, participants highlighted the inconsistent implementation of international frameworks regulating the transport of infectious substances. Although most countries had ratified the European *Agreement concerning the international carriage of dangerous goods by road*, the Biological Weapons Convention and the Nagoya Protocol,^12^^,^^16^^,^^18^ national incorporation of these frameworks remained uneven. In several Balkan states, redundant permits and overlapping jurisdictions caused delays of up to three weeks, whereas in Central Asia, approval could take two months. Some countries still relied on environmental or customs codes rather than dedicated biosafety laws, which led to conflicting definitions of infectious materials and unnecessary administrative burdens. Kazakhstan’s new biosafety law,^32^ for example, which integrates biological risk management into national security strategy, offers a promising model, yet most neighbouring countries remain without equivalent legislation or appropriate funding. In addition, dual-use export controls were inconsistently applied, further complicating legitimate diagnostic and research exchanges.

The IHR (2005) require the safe handling and timely movement of infectious substances but many WHO Member States lack harmonized rulebooks, standard operating procedures and clear enforcement mechanisms.^1^ These regulatory gaps create uncertainty and delay cross-border shipments, thereby undermining disease outbreak detection and regional health security.

### Institutional and capacity gaps

Ministries of health, transport, agriculture and finance often act independently of each other, have unclear mandates or lack coordination. Customs officers and laboratory personnel are frequently not trained in biosafety or infectious substance shipment, and few countries maintain certification frameworks for shippers or handlers of dangerous goods. Over 60% of workshop participants reported shortages of trained staff, appropriate packaging or authorized couriers for Category A materials.^3^^,^^4^

Limited infrastructure compounds these issue, particularly unreliable access to shipping material and/or cold-chain storage. Moreover, few governments allocate dedicated budgets for infectious substance shipment, relying instead on donor or emergency funds, thereby leaving capacity gaps unaddressed.

### Governance and coordination challenges

Poor communication between national authorities remains a challenge, with duplicate permit requests and unclear lines of responsibility causing delay and confusion. In Central Asia, the absence of Russian-language translations of WHO transport guidance limits access to current technical information. Workshop participants called for permanent coordination mechanisms, such as national liaison offices for infectious substance transport.

The absence, or inconsistent application, of biosafety and biosecurity laws remains a major vulnerability. Only a few workshop participants reported comprehensive frameworks for biosafety and biosecurity, whereas others relied on outdated or fragmented rules. Dual-use export control lists are often not updated in line with international standards, resulting in delays or the denial of cross-border shipments. Participants emphasized the need to align national legislation with international legislation to facilitate the safe and timely movement of samples.

Governance systems for infectious substance transport remain underdeveloped in many countries. Many have not designated a competent authority or established interministerial committees, while authorized shipper registries, national pathogen lists, digital tracking systems and standardized procedures for training, documentation, permits and incident reporting are incomplete or missing.

### Implications of the WHO Pandemic Agreement

The WHO Pandemic Agreement makes these operational questions legally salient.^2^ Its provisions on supply chains and logistics, on preparedness and resilience, and on the protection of health and logistics workers all presuppose that biological materials can cross borders quickly and lawfully.^2^ The pathogen access and benefit-sharing system established under Article 12 of the Agreement depends directly on efficient transport, since a system for the rapid sharing of pathogens with pandemic potential cannot operate faster than the permit and packaging processes and couriers involved. However, the precise obligations of that system remain open because the pathogen access and benefit-sharing annex of the WHO Pandemic Agreement was not adopted by the Seventy-ninth World Health Assembly in May 2026. Negotiations were extended within the Intergovernmental Working Group on the Pandemic Agreement, whose seventh session closed in July 2026;^42^ the outcome will be submitted to the Eightieth World Health Assembly in May 2027 or to an earlier special session.^43^ This interval is an opportunity rather than a delay. The national instruments identified as missing (that is, a designated competent authority, standard operating procedures, an authorized shipper registry and a time-bound permit pathway) are the same instruments through which any pathogen access and benefit-sharing system will have to operate. These can be built now, independently of how the annex is finally worded.

Several countries represented at the two workshops had already taken action. In 2022, Kazakhstan’s law on biological safety consolidated previously scattered rules, designated responsible authorities and placed biological risk management within the national security framework,^32^ which gave transport decisions a single legal reference point. Kyrgyzstan adopted comparable legislation in 2025.^44^ At the regional level and beyond, the *Agreement concerning the international carriage of dangerous goods by road* demonstrates what regulatory harmonization can achieve in practice: a consignment prepared once to a common standard is accepted across all contracting parties without renegotiation at each frontier.^12^ At the operational level, the WHO shipment mechanisms described above show that a centrally contracted courier and a single standing booking procedure can compress a shipment’s timescale from months to days.^39^^–^^41^ Although none of these actions provides a complete answer, each demonstrates that current bottlenecks are tractable.

A synthesis of discussions at the workshops revealed six priorities (Box 1): (i) implement national legislation harmonized with international transport and biosafety standards; (ii) establish coordination mechanisms for infectious substance transport and legal mandates for competent authorities; (iii) develop, implement or strengthen procedures, policy documents and standard operating procedures for interagency collaboration and coherency; (iv) institutionalize training and certification systems for shippers and customs officers; (v) integrate biosafety, dual-use and transport governance into preparedness planning and training; and (vi) develop digital permit and tracking systems to streamline processes. Box 1 provides further details, such as the actors best placed to implement the priority, and its principal advantage and cost.

Box 1 **Emerging policy priorities for international transport of infectious substances**

1. Implement national legislation harmonized with international transport and biosafety standards. This action addresses the finding that several states regulate infectious substances through environmental, veterinary or customs legislation that applies inconsistent definitions. Ministries of health, transport and justice should jointly review and align existing frameworks, preferably by amending regulations to ensure they reference the current edition of the UN Model Regulations on the Transport of Dangerous Goods^7^ and by assigning clear responsibilities for implementation, enforcement and periodic review. Legislative alignment can be slow and may require parliamentary action; once completed, however, it establishes consistent definitions and procedures for subsequent shipments and helps national requirements remain aligned with biennial revisions of international rules.
2. Establish coordination mechanisms for infectious substance transport and legal mandates for competent authorities. Many countries have never formally designated an authority for infectious substance transport. A government decree naming a lead authority and interministerial focal points is inexpensive and can usually be issued without primary legislation. Its weakness is that a mandate without dedicated staff and a budget becomes nominal, as reported by participants of several existing committees.
3. Develop, implement or strengthen procedures, policy documents and standard operating procedures for interagency collaboration and coherency. This action addresses the procedural ambiguity that was reported in both WHO subregions.^3^^,^^4^ National reference laboratories and customs administrations are the practical authors, with WHO and regional bodies supplying model texts. Standard operating procedures can be produced within months and shorten permit cycles immediately, but they are only as durable as the staff trained to use them and require scheduled revisions.
4. Institutionalize training and certification systems for shippers and customs officers. As certification is at present held by individuals rather than institutions, capability leaves with the post-holder. Embedding shipping competence in national laboratory and customs curricula, with periodic recertification, distributes competence across the system. Although the recurrent cost is real, the alternative of ad hoc external training is more expensive per person trained and competence does not accumulate.
5. Integrate biosafety, dual-use and transport governance into preparedness planning and training. Transport is treated as a logistics afterthought in most national action plans. Including it in International Health Regulations (2005) benchmarking, simulation exercises and after-action reviews adds little cost, as those exercises are already being conducted, and exposes bottlenecks before an emergency occurs. Inclusion requires health, security and customs sectors to plan jointly, which is where such integration most often stalls.
6. Develop digital permit and tracking systems to streamline processes. Paper-based, in-person permitting was cited by workshop participants as a primary cause of delay. Existing single-window platforms used by customs can often absorb this function. Digitalization offers the largest time saving of the six priority actions, but it is also the most capital-intensive and the most dependent on the other actions; automating an incoherent procedure only reproduces the incoherence faster.

WHO: World Health Organization; UN: United Nations.

The priorities are not equally achievable. Priorities 2 and 3 are administrative, can be delivered within a single budget cycle by existing institutions and are where most countries should begin. Priorities 4 and 5 require recurrent financing and a training infrastructure. Priorities 1 and 6 require legislative time and capital investment, respectively, and realistically are multiyear undertakings. However, sequencing matters: a designated authority operating under clear procedures makes subsequent legislative harmonization and digitalization straightforward, whereas digital systems built before roles are settled tend to encode existing fragmentation. Regional bodies and WHO can reduce the cost of less urgent priorities by supplying model legislation and shared training curricula and, where national transport volumes are too low to sustain a domestic market, by pooling procurement of certified packaging and dry ice.

To conclude, the international transport of infectious substances is governed by multiple overlapping regulatory frameworks whose inconsistent implementation creates delays, administrative burdens and barriers to public health collaboration. Weak institutional coordination, unclear responsibilities, and limited technical and operational capacity in many countries hinder safe and timely shipment. Harmonizing legislation, clarifying institutional mandates and establishing coordinated operational procedures would improve compliance with the IHR (2005), support implementation of the WHO Pandemic Agreement and strengthen preparedness for future health emergencies.
